# MicroRNA-Mediated Silencing Pathways in the Nervous System and Neurological Diseases

**DOI:** 10.3390/cells11152375

**Published:** 2022-08-02

**Authors:** Christian Barbato

**Affiliations:** Institute of Biochemistry and Cell Biology, National Research Council (CNR), Department of Sense Organs, University of Rome Sapienza, Policlinico Umberto I, 00161 Roma, Italy; christian.barbato@cnr.it

MicroRNAs (miRNAs) are small noncoding RNAs that play a prominent role in post-transcriptional gene regulation mechanisms in the brain tuning synaptic plasticity, memory formation, and cognitive functions in physiological and pathological conditions [1]. miRNAs are fine regulators expressed at different levels and in a neuronal-specific-type manner, and in a spatially and temporally controlled manner in the nervous system. Argonaute proteins are a core component of effector complexes (RISC/miRNP complex) of silencing mechanisms by miRNAs. The RNA-induced silencing complex (RISC), composed of Ago-2 and miRNAs, inhibits the target mRNA translation by an imperfect pairing between miRNAs and the 3′UTRs of the mRNA targets [2]. In biomedical and clinical research, miRNAs are important molecules in diagnostic and therapeutic strategies with special emphasis on neuroscience and neurological disease [3].

In this Special Issue are presented important findings on MicroRNA-Mediated Silencing Pathways in the Nervous System and Neurological Diseases in a series of publications. The basic mechanism of cooperation between miRNA, RNA-binding protein, and Ago2 silencing mechanism is investigated in neurons showing a novel finding that SERBP1 modulates Ago2/miR-92-mediated KCC2 regulation in neuronal cells [4]. From a future perspective might be interesting to explore the role of SERBP1/Ago2 complex and miR-92 on KCC2 regulation in Rett syndrome and other neurological disorders. In a study from Austria, Stojanovic et al. combined biophysical and pharmacological approaches to evaluate the miRNA-132/212 gene-deletion and nicotine stimulation age-dependent effect on synaptic plasticity in the mice hippocampus [5]. In addition, they investigated the effects of miRNA-132/212 gene-deletion in an established electrophysiological model of ischemia, indicating the miRNAs and oxygen-glucose deprivation are connected [6]. Two manuscripts from Di Pietro’s lab focused on miRNAs expression profile combined with proteins, in the late stage of Alzheimer's Disease, suggesting early Rap1 signaling activation [7]. Moreover, they confirm, as reported in the literature, the differentially expressed miRNAs and mutual signaling pathways, adding new unexplored interactions between microRNA and protein targets. The co-expression network analysis of miRNAs and proteins in the Alzheimer’s brain are reviewed in the second contribution [8]. In a study from Song lab, Kim et al. measured miR-1273g-3p in plasma and cerebrospinal fluid from AD patients. miR-1273g-3p enhanced amyloid beta production by inducing oxidative stress and mitochondrial impairments in AD model cell lines suggesting that miR-1273g-3p could be a candidate biomarker for early diagnosis of AD [9].

The potential pathophysiological mechanism and translational approaches to miRNA-mediated silencing pathways in neurological diseases are discussed in five excellent review articles. Liu et al. recapitulate advancements in the study of the roles of miRNAs in Amyotrophic lateral sclerosis (ALS) pathogenesis and its application to gene therapy [10]. Thomas and Zakharenko present the existing research evidence regarding the possible role of miRNAs in the pathology of schizophrenia [11]. Florian et al. attempt to collate the experimental evidence regarding microRNA and brain arteriovenous malformations (BAVMs) and cerebral cavernous malformations (CCMs) and other cerebral pathologies, showing a selected pool of associated miRNA [12]. Campos Pereira updates more recent studies on miRNA deregulation in prion disease, elucidating a translational approach from molecular to miRNA-based therapies, as frontier research in neurodegenerative disease [13]. Lastly, Blount et al. close this Special Issue with a critical review on the topic with a focus on miRNAs involvement in cognitive processes and dementia, mentioning the last suggestion generated by a new clinical frame of COVID-19 and SARS-CoV-2 virus [14].

The present Special Issue demonstrates advancement in the understanding of how miRNA works in the brain from studies on miRNA expression profile signatures from pathological and normal tissues in animal models and patients. An emerging research flow of in vitro and in vivo investigations of miRNA biological functions in neurogenesis, neurodevelopment, differentiation, axon morphogenesis, dendritic spine development, synaptic plasticity, and local protein synthesis prompts a molecular and cellular neurobiological exploration of the miRNA-mediated gene silencing in the nervous system.

Recent research works indicate two emerging aspects of microRNA-mediated silencing pathways: (i) the mechanisms of miRNA modification called tailing and trimming, and turnover [15] and (ii) that Argonaute proteins interact with a plethora of protein-binding partners [16]. The combination of this new evidences from basic research on microRNA biology, potentially integrated with significant progress that has been made in our understanding of miRNAs in the nervous system, as described in this Special Issue, provides an encouraging starting point to investigate miRNA pathway involvement in the development and progression of neurological and psychiatric diseases and to search future therapeutic applications.

This Special Issue is dedicated to Nadia Canu, who passed away on 19 May 2022. I was lucky enough to take my first steps in neurobiology, studying the Tau protein and the molecular and cellular mechanisms of Alzheimer's, in her laboratory from 1998 [17] to 2004 [18]. The lab's personal memories and anecdotes reflect her extraordinary confidentiality, a rare and uncommon quality. Nadia was a most extraordinary person and a brilliant teacher and scientist. She influenced my thinking and my career profoundly, and she will continue to live in our hearts and minds forever.
